# The Role of Artificial Intelligence in Modern Analytical Chemistry: Current Trends and Future Directions

**DOI:** 10.1155/ianc/2645726

**Published:** 2026-06-12

**Authors:** Samar H. Elagamy, Hemanth Kumar Chanduluru, Reem H. Obaydo, Hayam Mahmoud Lotfy

**Affiliations:** ^1^ Department of Pharmaceutical Analytical Chemistry, Faculty of Pharmacy, Tanta University, Tanta, 31111, Egypt, tanta.edu.eg; ^2^ SRM College of Pharmacy, Faculty of Medical and Health Sciences, SRM Institute of Science and Technology, Kattankulathur Chengalpattu, Tamil Nadu, 603203, India, srmist.edu.in; ^3^ Department of Analytical and Food Chemistry, Faculty of Pharmacy, Ebla Private University, Idlib, Syria; ^4^ Department of Pharmaceutical Analytical Chemistry, Faculty of Pharmacy, Cairo University, El-Kasr El-Aini Street, Cairo, 11562, Egypt, cu.edu.eg

**Keywords:** artificial intelligence (AI), deep learning (DL) machine learning (ML), method development

## Abstract

The integration of artificial intelligence (AI) plays a crucial role in modern analytical chemistry, offering solutions to long‐standing challenges. Conventional techniques, such as spectrophotometric analysis and chromatography, often face issues like spectral overlap, matrix interference, and extensive experimental optimization. AI and machine learning (ML) approaches address these limitations by enabling spectral deconvolution, pattern recognition, prediction of retention factors, and automated optimization of separation conditions. Beyond enhancing traditional methods, AI supports the development of innovative analytical platforms. Modern analytical chemistry increasingly relies on smartphone‐ and paper‐based sensors for on‐site detection of biomarkers and pollutants. These portable, low‐cost systems generate complex datasets requiring advanced computational tools, where AI can improve reliability and sensitivity when validated. AI also plays a vital role in synthesizing and optimizing nanomaterials such as carbon quantum dots (CQDs), accelerating experimental fine‐tuning through predictive modeling and optimization algorithms. Moreover, AI facilitates the interpretation of large‐scale data, providing deeper insights while reducing human error and analysis time. Despite these advancements, challenges remain regarding model interpretability and the integration of heterogeneous datasets. Addressing these requires explainable ML methods that bridge computational predictions with chemical reasoning. This review highlights current AI applications in chromatographic analysis, drug stability studies, and modern analytical chemistry, discusses implementation challenges, and explores future trends shaping the next generation of intelligent analytical systems.

## 1. Introduction

Over the past decade, artificial intelligence (AI) has become a driving force in drug discovery [1–4] and genomics [5–10], enabling tasks such as virtual screening of drug candidates, prediction of molecular properties, identification of novel therapeutic targets, and interpretation of large‐scale genomic data with increased speed and often improved predictive performance when trained and validated appropriately [11, 12]. In recent years, AI and machine learning (ML) have emerged as powerful tools in analytical chemistry, with rapidly growing applications in pharmaceutical analysis [13]. The determination of pharmaceuticals in diverse and complex matrices often generates vast datasets, which pose significant challenges for conventional analytical methods. In this context, AI‐based strategies provide advanced capabilities for experimental design, method optimization, and data interpretation [14].

AI algorithms can process large and heterogeneous datasets, extracting patterns that can potentially improve accuracy, reliability, and reproducibility when models are trained on representative datasets and externally validated. This capability has accelerated the development of analytical methods by supporting predictive modeling, optimization of experimental parameters, and material property prediction [15–18].

With the continuous advancements of analytical methods, such as smartphone‐assisted methods [19–21], the integration of nanomaterials [22–25], and the rise of multimodal and multiarray approaches [26–28], AI implementation has become increasingly essential. AI also has significant applications in electrochemical sensing, where it enhances electrochemical peak resolution and lowers detection limits in voltammetric analysis, particularly for complex and multiplexed real‐sample matrices [29]. For effective implementation of AI in method development, it requires access to large, high‐quality datasets for model training and improved interpretability of computational outputs.

Several review articles have addressed the role of AI in analytical chemistry. For example, Rial in Talanta (2024) provided an overview of AI‐driven methodologies in analytical workflows, focusing on data processing strategies and emerging digital tools [30]. Similarly, Debus et al. in TrAC Trends in Analytical Chemistry (2021) presented a comprehensive review of deep learning (DL) approaches in analytical chemistry, highlighting neural network architectures and their performance in spectral and chromatographic data analysis [31]. In addition, Houhou and Bocklitz discussed recent trends in AI, ML, and chemometrics applied to chemical data, with particular emphasis on algorithm development and pattern recognition techniques [32]. Unlike these previous reviews, the present review provides a more comprehensive and application‐oriented perspective. It systematically covers AI applications across multiple analytical domains, including chromatography, spectroscopic analysis, nanomaterial synthesis, and drug stability studies. This review provides a comprehensive overview of AI applications in modern analytical chemistry, focusing on method development, optimization, and data interpretation, with dedicated sections on chromatography and drug stability. It also highlights the utility of specific tools and datasets, such as the YesChat HPLC Method Developer and the Genentech Multi‐Column Retention Time (GMCRT) dataset, as well as current limitations and challenges associated with AI adoption. Furthermore, it outlines future perspectives, emphasizing the transformative potential of AI in advancing sustainable, precise, and intelligent analysis.

### 1.1. Conceptual Background: AI and Related Approaches

AI is a branch of computer science dedicated to creating systems capable of performing tasks that traditionally require human intelligence, such as reasoning, learning, and perception [32]. Generally, AI has been applied to automate data interpretation and optimize experimental design. ML, a subset of AI, focuses on algorithms that learn from data and enhance performance over time without explicit programming for each task [30].

ML is widely employed to process large datasets obtained from conventional analytical methods such as spectrophotometry and chromatography, where it enables the prediction of results [33–35]. ML models can be trained using three primary approaches: supervised, semisupervised, and unsupervised learning, depending on the availability of labeled data. In supervised learning, training datasets contain both input and output pairs, allowing the algorithm to learn a mapping function that accurately predicts outputs from new inputs. Unsupervised learning, in contrast, is applied when only input data are available, focusing on uncovering hidden structures, patterns, or clusters within the dataset [36]. Semisupervised learning serves as an intermediate approach, proving particularly useful when abundant input data exists but only a limited portion of the output data is labeled [34]. Among these strategies, supervised learning is the most advanced and widely applied in analytical chemistry, as it enables precise predictions based on well‐annotated data [37, 38].

ML approaches in analytical chemistry can also be grouped into three principal categories [34, 37].

First, exploratory analysis aims to reveal latent structure within complex datasets without predefined labels. Techniques such as unsupervised learning techniques, principal component analysis (PCA), hierarchical clustering analysis (HCA), and t‐distributed stochastic neighbor embedding (t‐SNE) are commonly used to visualize clustering patterns, detect outliers, assess batch effects, and monitor instrumental drift.

Second, qualitative classification focuses on identification and discrimination tasks, including compound recognition, authenticity assessment, and screening decisions. Representative approaches include supervised learning techniques such as partial least squares–discriminant analysis (PLS–DA), support vector machines (SVMs), random forests (RFs), and convolutional neural networks (CNNs).

Third, quantitative modeling and calibration involve predicting continuous outputs such as concentration, retention time (RT), degradation extent, or physicochemical properties. Methods such as partial least squares (PLS), support vector regression (SVR), RF regression, and multilayer perceptrons (MLPs) are widely employed.

DL represents a further specialization of ML, based on multilayered artificial neural networks (ANNs) capable of extracting hierarchical data representations [31]. This approach is efficient for analyzing large‐scale and complex datasets, including images and spectra. For example, deep neural networks (DNNs), CNNs, recurrent neural networks (RNNs), and auto‐encoders (AEs). DL has been successfully applied to spectral deconvolution and image interpretation. Unlike traditional ML, which often requires manual feature extraction, DL can automatically learn features and achieve higher accuracy, minimizing human intervention thanks to its greater computational power [39, 40].

Chemometrics, meanwhile, involves extracting chemical information through statistical and mathematical approaches. It is commonly applied to experimental design, process optimization, and method validation, often using techniques such as regression and PCA. While traditional chemometrics emphasizes interpretable statistical models, the integration of AI and ML enables the handling of nonlinear and complex problems, thereby expanding its analytical power [32, 41–43].

Although AI algorithms provide powerful capabilities for analyzing complex data, their “black box” nature can limit confidence in their outputs, particularly when models are used to support analytical decisions. Accordingly, increasing attention is being directed toward explainable ML approaches that improve transparency and connect computational outputs to chemical interpretation. Representative examples include Shapley additive explanations (SHAP) and explanation approaches for neural networks (e.g., class activation mapping [CAM] in convolutional models), which can help identify influential variables or signal regions and support chemically plausible interpretation [44–49]. Their integration promises to refine analytical methodologies, improve trust in AI systems, and accelerate innovation across spectrophotometry, chromatography, and pharmaceutical research.

### 1.2. Applications of AI‐Based Approaches

**FIGURE 1:**
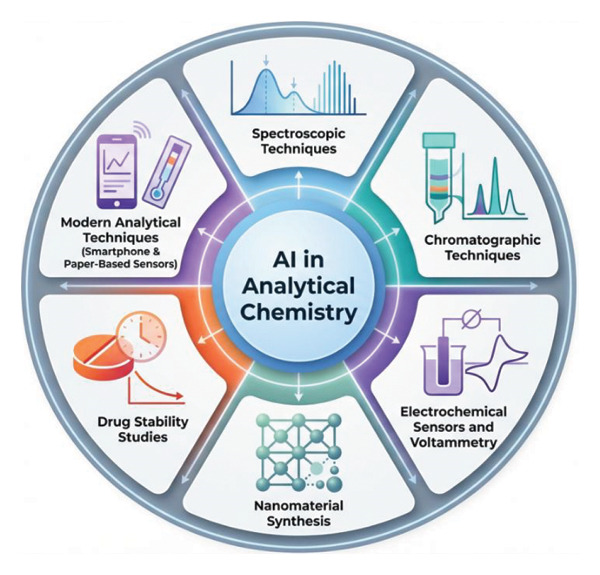
Diverse applications of AI across different aspects of analytical chemistry.

**FIGURE 2:**
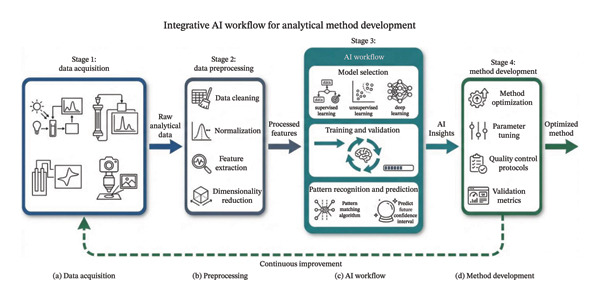
Integrative AI workflow for analytical method development.

AI‐based approaches have shown strong potential in analytical chemistry by enabling quantitative analysis, compound identification, spectral interpretation, image analysis, and optimizing material synthesis. Their power lies in extracting complex patterns from large datasets and addressing challenges associated with biological, medical, and environmental sample matrices. Figure 1 illustrates the diverse application areas of AI across analytical chemistry. To connect these applications with practical method development and routine use, Figure 2 summarizes an integrative workflow spanning the definition of the analytical target profile (ATP), experimental design, and data acquisition with metadata capture, analytical preprocessing and QA/QC, model selection, and method development. The following sections provide a detailed discussion of the applications of AI and the associated good‐practice considerations.

#### 1.2.1. Applications in Spectroscopic Techniques

Spectroscopic data often suffer from issues such as baseline drift, noise, peak shifts, peak overlaps, and instrument‐related artifacts, making the resolution, identification, and quantification of target compounds difficult. Preprocessing techniques, such as noise reduction, baseline correction, scaling, and weighting, are essential but should not substitute for a well‐designed experiment. Data quality, accurate annotation, and balanced datasets are critical for reliable AI models. Mislabeling, unbalanced datasets, and the recognition of rare events remain significant challenges [50–52].

AI algorithms have been successfully applied in ultraviolet–visible (UV–vis) spectrophotometry and FTIR analysis, with numerous applications that help correlate spectral features for improved classification and identification [53, 54]. Table 1 presents examples of ML models applied in spectroscopic analysis. Among these, supervised ML models are the most commonly employed. Different models are utilized, with their performance typically evaluated using correlation coefficients and root mean square error (RMSE). Unsupervised methods, including dimensionality reduction techniques such as PCA, are also employed. Training ML models often requires large amounts of experimental data, as seen in Table 1.

**TABLE 1:** Examples of applications of ML models in spectrophotometric techniques.

Technique	AI model	Dataset/input description	Analytical objective	Application domain	AI contribution to study	Ref
UV–vis spectrophotometry	Support vector regression (SVR)	UV–vis spectra (200–800 nm) of 40 honey samples; random split 70%/30% (28 calibration, 12 validation); spectra averaged per sample (quadruplicate mentioned)	Quantitative prediction of sucrose content in honey	Food analysis/quality control	Multivariate regression enabling concentration prediction from full spectra as a rapid screening approach	[55]
UV–VIS spectrophotometry	OPLS‐DA; Support vector machine (SVM) (with PCA exploratory analysis)	162 UV–VIS spectra (45 wines in triplicate + 27 QC); split by Kennard–Stone 80%/20% into training (109) and prediction (26) sets	Classification of Chinese red wines by geographical origin	Food authentication/origin traceability	Discrimination of similar spectral profiles; QC samples used to monitor measurement stability	[56]
UV–VIS spectrophotometry	Random forest (RF)	UV–VIS peak lists (λmax and MEC) for 74,784 molecules in methanol; labeled POS/NEG by ICH S10 (290–700 nm, MEC ≥ 1000); split into training 72,788 and two external test sets (998 + 998)	Classification of compounds by photoreactive potential (POS/NEG)	Photosafety/Photochemical assessment	Large‐scale structure⟶ UV–vis feature classification with two independent external test sets	[57]
FTIR	LDA; KNN; SVM (with PCA and SNV; LOOCV)	‐FTIR spectra of PVA/PVP blend films (PVP < 3 wt%); five concentrations (0–3 wt%); 30 samples (duplicate) per concentration; each sample measured at 3 spots per duplicate; LOOCV validation	Classification of low‐concentration polymer blends	Polymer science/materials characterization	Classification at very low concentration changes using preprocessing, spectral‐range choice, and simple classifiers	[58]
FTIR	RF; GB; DT; k‐NN; LR; SVM; MLP	FTIR spectra of undegraded vs aged PET microplastics; models optimized using 5‐fold CV; evaluated using confusion matrix and precision/recall/F1	Classification of the PET microplastics aging state	Environmental monitoring/microplastics analysis	Automated identification of degradation‐related spectral differences using ML with cross‐validation and multimetric reporting	[59]
FTIR	KNN; LR; SVM; DT; RF (RF best; PCA used)	1071 seized drug samples analyzed in triplicate ⟶ 3213 FTIR spectra; GC–MS‐confirmed labels; PCA retained 70% variance; train/test 70%/30%; additional test on new unseen samples reported	Classification of methamphetamine, heroin, and benzodiazepines	Forensic analysis	Rapid multiclass classification of forensic spectra using ML supported by confirmed reference labels	[60]
FT‐IR	SVM; RF; LDA (classification) + SVR/PLS (regression also reported)	FTIR matrix 184 samples × 540 variables; train/test 75%/25% (balanced test set); SVM tuning by 5‐fold CV on training set	Detection and quantification of juice adulteration	Food authentication/Adulteration detection	Full‐spectrum “spectral print” modeling with an external test set for robust classification and regression	[61]

*Note:* KNN/k‐NN, k‐nearest neighbors; MLP, multilayer perceptron; F1, F1‐score; POS/NEG, positive/negative class labels; PET, polyethylene terephthalate; Reaxys, chemical literature database used for data mining.

Abbreviations: CV, cross‐validation; DT, decision tree; GB, gradient boosting; GC–MS, gas chromatography–mass spectrometry; ICH, International Council for Harmonisation; LDA, linear discriminant analysis; LOOCV, leave‐one‐out cross‐validation; LR, logistic regression; MEC, molar extinction coefficient; OPLS‐DA, orthogonal partial least squares–discriminant analysis; PCA, principal component analysis; QC, quality control; RF, random forest; SVM, support vector machine; SVR, support vector regression.

It should be noted that AI approaches, besides assisting in resolving overlapped spectra and their interpretation, also contribute to the development and optimization of spectrophotometric methods. Lotfy et al. utilized Microsoft Copilot, an open‐access AI‐powered tool, in developing a spectrophotometric method for determining the solifenacin–mirabegron combination. Microsoft Copilot proved effective in optimizing wavelength selection by evaluating recovery and precision metrics, analyzing recorded absorbance values, and assessing efficiency with minimal variability [62]. AI facilitates spectral optimization by identifying wavelength pairs that maximize accuracy and precision while minimizing signal deviation. However, its recommendations are inherently limited to the provided dataset, relying strictly on recorded absorbance signals without external validation. To confirm AI‐derived adjustments, AI‐driven optimization should be accompanied by risk analysis using cumulative validation scores that incorporate essential parameters such as accuracy, precision, and robustness, along with experimental verification and statistical validation.

**FIGURE 3:**
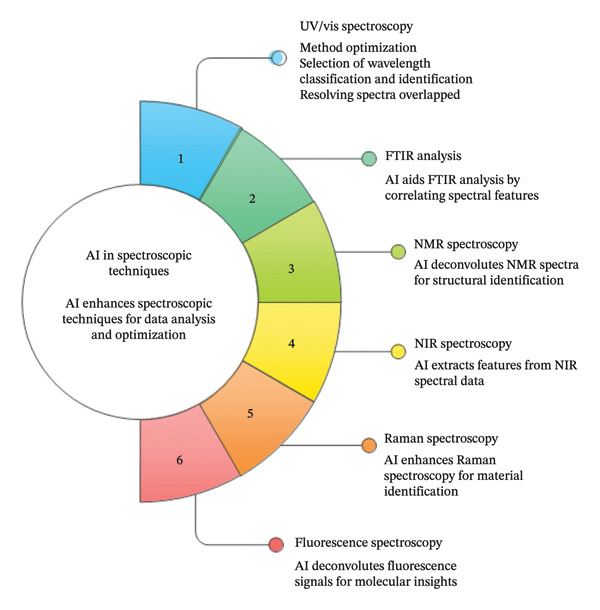
Summary of the role of AI in different spectroscopic techniques.

ML has been successfully integrated with other spectroscopic techniques, such as nuclear magnetic resonance (NMR) [63], near‐infrared (NIR) analysis [64–67], Raman [68–70], and fluorescence [71, 72]. The role of AI in different spectroscopic techniques is summarized in Figure 3. In NMR, AI algorithms have been used to deconvolute complex spectra, allowing rapid and accurate structural identification [73]. In NIR analysis, ML algorithms have been introduced to extract standard features from spectral data [67, 74]. Raman spectrophotometry has also advanced through AI integration, where ML models improve the interpretation of spectra for material and compound identification in diverse applications ranging from environmental monitoring to forensic science [75–81]. In fluorescence measurements, ML algorithms are applied to deconvolute overlapping signals from multiple fluorophores, enabling more profound insights into molecular composition and biochemical dynamics [82, 83].

A remarkable example of explainable ML in spectroscopic analysis was presented by Wang et al., who developed an ML‐based predictive model for estimating haloacetic acids (HAAs) concentrations in secondary water supply systems using UV–vis absorption and excitation–emission matrix (EEM) fluorescence analysis. Among the tested approaches, the RF model achieved the best predictive performance, which was further enhanced by integrating spectral data with additional water quality parameters. To improve interpretability, the authors employed SHAP analysis, a method that quantifies both the magnitude and direction of each variable’s contribution to the prediction [84].

The dataset was divided into a training set (80%) and a testing set (20%), ensuring robust validation. SHAP revealed which spectral and water quality features most strongly influenced HAA predictions, distinguishing between positive and negative contributions while ranking their relative importance. By integrating these quantitative insights with domain knowledge, the study demonstrated how explainable ML approaches can both maximize prediction accuracy and enhance reliability in water quality monitoring. Another example of explainable ML models was presented by Kalatzis et al., who focused on classifying pharmaceutical compounds based on Raman spectra. Using an open‐source dataset of 3510 spectra from 32 active pharmaceutical ingredient (API)–related compounds, covering the range 150–3425 cm^−1^, the study evaluated both traditional models, such as SVMs, RF, and k‐nearest neighbors (k‐NN), and a DL model in the form of a one‐dimensional convolutional neural network (1D CNN). A SHAP‐based explanation was applied to interpret model outputs, revealing which vibrational modes were most influential in distinguishing between compounds [85]. This approach confirms the growing importance of interpretable AI tools in pharmaceutical analysis and quality control, where transparency in decision‐making is as crucial as predictive accuracy.

AI has significantly improved spectrophotometric analysis by enabling more accurate spectral data analysis and interpretation. ML algorithms can quickly identify complex patterns and assign spectral features, reducing analysis time. Interpretability of AI decisions remains a challenge, potentially limiting trust in automated results and the need for monitoring by an expert analyst.

#### 1.2.2. Applications in Chromatographic Techniques

The applications of AI algorithms in chromatography involve processing and evaluating large chromatographic datasets, identifying patterns and correlations within complex information [18]. This enables more accurate identification and quantification of compounds.

In chromatography, AI may be applied at two distinct data levels. Signal‐level approaches treat the entire chromatogram (detector response vs time) as a numerical vector to support baseline correction, peak detection/deconvolution, and pattern recognition. Feature‐level approaches use extracted chromatographic outputs such as RTs, peak areas/heights, peak ratios, or calculated concentrations as model inputs for classification, quantification, or method optimization.

**FIGURE 4:**
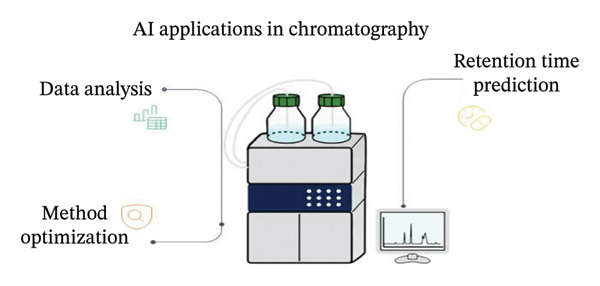
Summary of the role of AI in chromatographic techniques.

Beyond data analysis, AI also plays a vital role in optimizing chromatographic methods and processes. Algorithms can predict the most suitable experimental conditions for separating specific compounds, such as the ideal mobile phase (MP) composition, gradient design in LC, and temperature optimization. Temperature programming is particularly critical in gas chromatography (GC), where AI‐driven models can assist in designing optimal heating ramps to improve resolution, reduce analysis time, and enhance peak capacity. This leads to significant savings in time and resources, while improving reproducibility and efficiency [86–89]. The role of AI in chromatographic techniques is illustrated in Figure 4.

Lotfy et al. [90] utilized an AI‐based tool to develop an LC method for the simultaneous separation of amlodipine, hydrochlorothiazide, and candesartan. The tool, YesChat HPLC Method Developer, used as an LC method optimization tool, functions as a specialized GPT‐driven model trained on large datasets of published chromatographic methods and physicochemical properties of analytes. In this study, the input parameters provided included the chemical names and structures of the analytes, along with the design of a technique suitable for pharmaceutical analysis. Based on these inputs, the AI tool predicted the following initial chromatographic conditions: a C18 column (150 × 4.6 mm, 5 μm), gradient elution with 20 mM phosphate buffer (pH 3.0) and acetonitrile, a flow rate of 1.0 mL/min, and UV detection at 240 nm. The method was expected to achieve baseline separation with an estimated run time of approximately 15 min [90].

Recent advances in analytical chemistry have integrated AI tools such as Copilot, ChatGPT 5.2, Gemini, and Perplexity to enhance method development [90]. In this recent study, two (HPLC) methods were developed and validated for the simultaneous determination of dolutegravir, lamivudine, and abacavir in their pharmaceutical formulations. These AI‐driven approaches involved predicting optimal conditions, which were then experimentally refined, significantly improving efficiency and accuracy.

The first method is an isocratic HPLC using an XBridge C18 column with a MP of acetonitrile and phosphate buffer (pH 3.5), allowing simultaneous quantification of DTG and 3TC at detection wavelengths of 258 nm and 275 nm, respectively. The second method is a gradient HPLC employing a Spherisorb ODS2 C18 column with methanol and TEA/TFA buffer (pH 3.15), enabling the simultaneous analysis of all three drugs at 258, 278, and 294 nm. Both methods exhibit wide linear ranges, validated according to ICH Q2(R2) guidelines, and exemplify the integration of green and white analytical chemistry principles with AI for rapid, accurate, and sustainable routine quality control.

ML has emerged as an important tool in chromatographic techniques. Do et al. demonstrated this by constructing predictive models to estimate HPTLC retention factors (Rf) based on molecular chemical properties. Various regression algorithms, including SVMs, Rf, and linear regression, were trained using a dataset of 178 reference substances and subsequently applied to predict the Rf values of 20 additional compounds from diverse chemical classes. The study revealed that model performance strongly depends on the structural similarity between compounds in the training and test sets. This approach highlights the potential of computational methods to enhance the evaluation of HPTLC data, reduce experimental workload, and support more efficient method development [91].

AI‐based approaches have also been increasingly applied in chromatography in the prediction of RT, often in collaboration with quantitative structure–retention relationship (QSRR) modeling [92, 93]. QSRR is a powerful strategy in chromatographic analysis that describes the relationship between a solute’s RT and its physicochemical properties, which are represented numerically through molecular descriptors. These descriptors can be classified into different levels: 0D (derived from chemical formulas), 1D (substructural descriptors), 2D (topological descriptors), 3D (geometrical descriptors), and 4D (stereoelectronic descriptors), with 2D and 3D being the most widely used in QSRR studies. They can be calculated using specialized software packages that require molecular representations such as SMILES or InChI strings, usually retrieved from chemical databases [94, 95]. A recurring limitation of RT models is reduced performance under domain shift, such as changes in column chemistry, instrument setup, or mobile‐phase conditions, unless external validation or transfer strategies are included.

The most critical steps in developing ML models for RT prediction are the choice of input features that accurately reflect the chromatographic behavior of molecules [96]. Molecular descriptors are among the most commonly used features in chemistry. In addition, molecular fingerprints, which are binary bit strings representing the absence (0) or presence (1) of specific structural patterns, are frequently applied, particularly in similarity searching. Fingerprints can be generated using different strategies, including circular molecular fingerprints based on the local atomic environment, functional‐class fingerprints reflecting pharmacophoric properties, and those encoding hybridization and aromaticity [89, 97]. Representative sampling of molecules is also crucial in ML models, as it enhances the predictive ability and applicability of RT models. However, many studies rely on relatively small experimental datasets, often comprising only dozens or hundreds of molecules, or on open‐access datasets [98]. The major challenge with limited datasets is poor generalization: models trained on narrow chemical spaces may struggle to predict RTs for compounds that differ substantially from those in the training set.

An illustrative example of the effective implementation of ML in LC method development is provided by Moorthy et al., who applied ML‐based analysis to predict the RTs of pesticides, enabling eco‐friendly and cost‐effective analytical method design. A classification model was developed using two algorithms, SVM and Naïve Bayes, to assess the influence of structural features on the retention behavior of 324 pesticides. Physicochemical descriptors and Molecular ACCess System (MACCS) fingerprints were calculated using PaDEL software. Reported RTs were classified into two categories: > 21 min (high RT, poor eluting power) and < 21 min (low RT, good eluting power). The models were trained on the complete dataset and validated using a test set (30%) and fivefold cross‐validation, demonstrating strong performance through robust statistical parameters, including accuracy, precision, and specificity. Feature analysis revealed that structural elements, including a single six‐membered aromatic ring or = O‐containing rings, were associated with shorter RTs, while compounds with multiple aromatic rings, double bonds, sulfoxide groups, oxygen‐containing heterocycles, or sulfur‐heteroatom linkages exhibited longer RTs. Conversely, the presence of amino groups, phosphorus atoms, sulfur atoms, tertiary groups, or multiple methyl/methoxy substituents contributed to shorter RTs. These insights suggested that selecting solvents with higher polarity could accelerate elution, reducing RT for pesticides with such structural features [99].

This study demonstrated the ability of ML to capture complex, nonlinear relationships between molecular structures and chromatographic behavior, outperforming traditional QSRR approaches, which often struggle with datasets generated under nonuniform experimental conditions. By overcoming the noise and variability inherent in such datasets, ML‐based classification provided more reliable predictive models, supporting the development of efficient and sustainable LC methods.

It should be noted that Shi et al. developed the GMCRT dataset, which contains 1790 RT values of 51 small‐molecule pharmaceutical compounds collected across six Genentech clinical phase projects using 20 commonly employed RP‐HPLC columns. This dataset was used to train QSRR‐based ML models that incorporated both molecular descriptors of analytes and chromatographic method parameters. The database also included stationary phase (SP) selectivity descriptors from the hydrophobic subtraction model (HSM), namely, hydrophobicity (H), steric hindrance (S), hydrogen‐bond acidity (A), hydrogen‐bond basicity (B), ionic interaction (C), and the ethylbenzene (EB) Rf, under two pH conditions (2.8 and 7). Two ML approaches, PLS and ANNs, demonstrated improved accuracy in predicting RT on new SPs compared to previously published direct mapping strategies, particularly when RP‐HPLC columns exhibited significant selectivity differences. Unlike earlier methods, this approach does not require preexisting retention data on the target SPs. It is generalizable to any RP‐HPLC column, provided the HSM descriptors, MP pH, column dimensions, and flow rate are known. To further validate the models, an independent test set comprising 24 additional Genentech compounds from four development projects (144 RT values in total) was used, confirming the robust performance of the models. Finally, the GMCRT dataset was leveraged to demonstrate the generalizability of ML‐based RT prediction under unseen SP and MP conditions, underscoring its potential as a powerful tool in chromatographic method development [100].

Thus, AI optimizes chromatographic separation processes by predicting optimal conditions and improving peak resolution. AI assists in method development and troubleshooting, saving time and resources. Nonetheless, AI models may struggle with variability in samples and instrument conditions, affecting robustness. Overreliance on AI could lead to reduced understanding of underlying chemistry. Despite these challenges, AI enhances workflow efficiency and reproducibility in chromatographic analyses.

#### 1.2.3. Applications of AI in Electrochemical Sensors and Voltammetry

Electrochemical sensors are attractive for rapid and low‐cost analysis because the instrumentation can be compact and the signals are information‐rich. In practice, however, qualitative identification and semiquantitative interpretation can be challenging due to peak overlap between electroactive species, matrix effects (ionic strength, interferents, and real waters), platform‐to‐platform variability (especially with disposable electrodes), and electrode fouling/passivation, as well as operator/instrument variability. These factors can distort peak positions and peak shapes and reduce the reliability of classical peak‐picking and calibration strategies, motivating the use of ML and DL to improve robustness and decision‐making from voltammetric data [42].

**FIGURE 5:**
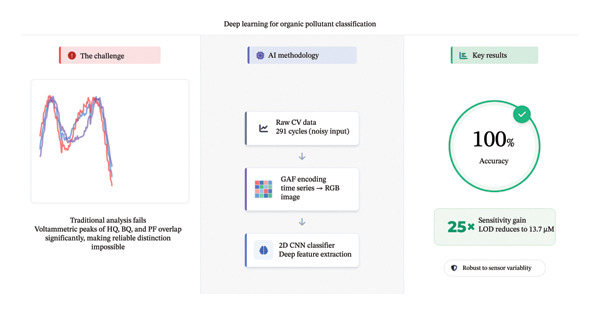
Example of the DL approach for organic pollutant classification shows overlapping CV curves. Scans are transformed into RGB images using GAF encoding and classified using a 2D CNN, enabling robust discrimination under peak overlap and sensor variability [101].

A key emerging direction is to treat voltammograms as high‐information “fingerprints” and use AI models capable of learning discriminative patterns under overlap and noise. Two complementary strategies are commonly used: feature‐level ML, where engineered descriptors (e.g., peak currents/potentials, integrated areas, reduced‐dimensional representations) are used as inputs, and signal‐level DL, where models learn directly from the full scan (time series) or from transformed representations of the scan. A practical example of signal‐level learning is time‐series‐to‐image encoding, such as the Gramian angular field (GAF), which converts voltammetric scans into image‐like representations so that two‐dimensional convolutional neural networks (2D CNNs) can exploit spatial feature extraction while preserving temporal relationships in the original signal. Figure 5 illustrates this concept using cyclic voltammetry (CV) data for organic pollutant classification: Noisy CV scans are encoded via GAF into RGB images and then classified using a 2D CNN, enabling discrimination of challenging analytes with overlapping voltammetric features [101].

Beyond prediction accuracy, several considerations largely determine whether voltammetry–AI pipelines are credible and transferable in routine analytical practice. Leakage‐safe validation is essential because repeated scans and closely related replicates can inflate apparent performance if split incorrectly. Domain‐shift robustness should be assessed because changes in electrode batch, instrument, operator, or matrix composition can shift the voltammetric fingerprint. Interpretability and chemical plausibility are also important; for example, CAMs can help confirm that a model focuses on chemically meaningful scan regions rather than artifacts. Table 2 summarizes representative voltammetry AI workflows across different voltammetric modalities, matrices, and model families, highlighting validation approaches.

**TABLE 2:** Representative AI workflows for electrochemical sensing (voltammetry).

What was measured and why AI was needed	Experimental setting (matrix + electrode)	AI approach	Main outcome + comment	Ref.
CV to distinguish hydroquinone vs benzoquinone with overlapping peaks	Buffer; disposable SPEs (bare and CNT‐modified)	GAF image encoding + CNN classification	100% accuracy (3 classes) was reported, but routine translation should be supported by leakage‐safe splitting and independent electrode‐batch/laboratory validation	[101]
Voltammetry for multiplex identification (individuals + mixtures) under peak overlap	Deionized and tap water; bare SPEs	GAF image encoding + CNN classification	Near‐perfect confusion matrices with limited confusion were reported, but generalization should be verified on larger datasets covering additional matrices and concentration designs	[29]
CSWV for chemical identification (hazardous chemicals and explosives)	Seawater (multiple sites; field measurements) and buffer; handheld potentiostat with SPEs	Joined reduction + oxidation scans into one curve; compared ML/DL classifiers; used CAMs	Very high performance was reported (ROC–AUC > 0.99 for several datasets) with good held‐out/new‐data results, but robustness should be rechecked under electrode/instrument changes and limited‐sample conditions	[102]

*Note:* CNT: carbon nanotube(s), ROC–AUC: area under the receiver operating characteristic curve.

Abbreviations: CSWV, cyclic square‐wave voltammetry; SPE(s), screen‐printed electrode(s).

AI enhances sensor technology by improving signal processing and data interpretation in real time. ML algorithms increase sensor sensitivity and selectivity, enabling detection of low‐concentration analytes. Challenges include the risk of overfitting models to specific conditions and difficulties in maintaining accuracy across diverse environments. There are also concerns about data privacy and security in AI‐enabled sensors. Despite these limitations, AI makes sensors more versatile, reliable, and suitable for various practical applications.

#### 1.2.4. Applications in Nanomaterial Synthesis

AI‐based approaches are emerging as crucial tools in the design and analysis of nanomaterials, revolutionizing the field of material engineering [103–105]. AI is increasingly utilized to predict material properties, enabling the design of novel materials and the discovery of underlying mechanisms. Pashkov et al. designed a supervised ML model for the structural analysis of gold nanoparticles Ag NPs using surface plasmon resonance, which is highly sensitive to nanoparticle size and shape. The model is based on quantitative analysis of UV–vis spectra of Ag NPs, which provide insights into the structural parameters of the nanoparticles, addressing the inverse design problem. The model was trained on theoretical datasets and applied to predict spectra for different combinations of structural parameters [106].

ML models provide a robust approach for unraveling the complex relationships between carbon quantum dots (CQDs) structures and their properties. ML models can be designed to incorporate a diverse range of descriptors as input features, including compositional information such as elemental ratios (C/N or C/O) and surface functional group distributions, as well as structural and morphological characteristics like particle size, shape, crystallinity, and defect density. Optical and electronic parameters, such as absorption and emission spectra, quantum yield (QY), photoluminescence lifetime, and bandgap energy, further enrich the input space. Meanwhile, spectroscopic fingerprints from Raman, FTIR, or XPS analysis provide detailed insights into bonding environments and surface chemistry. Additionally, synthesis‐related variables, such as precursor composition, reaction temperature, pH, and postsynthetic modifications, can be incorporated, offering additional context for property prediction. Incorporating these descriptors enables ML models to generate more accurate forecasts and deepen the understanding of the intricate factors governing CQD behavior and applications [107, 108]. Supervised learning models, for example, can predict CQD characteristics by leveraging large datasets that link molecular structures with their corresponding features. Such predictive models generate valuable insights into CQD behavior, supporting the design of customized applications that cater to individual needs. In parallel, unsupervised learning methods probe unexplored regions of the vast chemical space of CQDs. By detecting hidden patterns and correlations within data, these approaches reveal novel attributes and open pathways to the discovery of previously unrecognized properties and potential applications. In 2025, Kannoma et al. developed an ML‐based framework to design CQDs with high QY by examining the influence of various physicochemical parameters and precursor atomic compositions. A dataset was constructed by combining 117 data points from their own studies on CQD synthesis and QY, where each trial represented a unique combination of three precursors. In total, 2600 possible precursor combinations were considered, of which 117 were randomly selected for dataset construction. Each trial included 17 input features describing the precursors, such as elemental composition (%C, %H, %O, %N, %P, %S), polar and molecular surface areas (PSA and MSA), refractivity (REF), hydrogen donor and acceptor counts, rotatable bond count (RBC), fraction of sp^3^ carbon, polarizability, solvation energy, molecular polarizability anisotropy (MPA), and molecular polarizability ratio (MPR). The measured QY values of the prepared CQDs served as output labels. After dataset preprocessing, 6 ML models, including stochastic gradient descent (SGD), RF, AdaBoost, Bagging, SVR, and MLP, were trained and evaluated. The MLP regression model outperformed the others, achieving the lowest mean absolute error and RMSE, as well as the highest coefficient of determination. To further optimize synthesis conditions, a genetic algorithm (GA) was applied, as it is well recognized for efficiently solving complex optimization problems. The GA explored diverse precursor concentrations and microwave irradiation parameters (time and power), which are known to influence QY strongly. The RF model was selected as the predictive model due to its superior performance (*R* ^2^ = 0.9764, RMSE = 4.53), and GA‐optimized conditions were subsequently validated experimentally, yielding CQDs with a QY of 79% (close to the 83.19% predicted by the model) [109].

However, the application of AI and ML in this field of nanomaterial synthesis remains at an early stage and faces several critical challenges. A major limitation is the scarcity and inconsistency of available experimental data. Many studies on nanomaterial synthesis do not report key parameters in a standardized manner, including precursor composition, reaction temperature, reaction time, and postsynthesis treatments. Additionally, variations in characterization techniques and selective reporting of successful results introduce data bias, which reduces model reliability.

Another important barrier is the limited availability of open‐access datasets. Large, high‐quality datasets are essential for training and validating ML models, but the lack of centralized, openly accessible data hinders reproducibility and broader adoption. To overcome these challenges, the research community should establish standardized reporting protocols and develop comprehensive, open‐access databases.

Further limitations include the risk of model overfitting, where algorithms perform well on training data but fail to generalize to new experimental conditions. Many ML algorithms are also criticized for their limited interpretability, which makes it difficult for researchers to extract meaningful mechanistic insights from their predictions [110].

#### 1.2.5. Applications in Modern Analytical Techniques (Smartphone and Paper‐Based Methods)

Applications of AI algorithms in modern analytical techniques, such as smartphone‐based and paper‐based sensors, are rapidly advancing. ML algorithms are typically implemented in smartphone‐based workflows, facilitating the real‐time acquisition, correction, and display of colorimetric data [111, 112].

**FIGURE 6:**
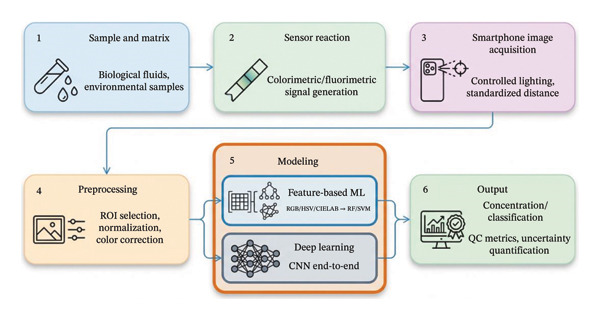
End‐to‐end smartphone/paper‐based workflow.

These smartphone‐assisted ML workflows can be implemented as end‐to‐end pipelines (Figure 6) that include sample collection, signal generation at the sensor (colorimetric/fluorimetric reaction), smartphone image capture, image preprocessing (selecting the region of interest and correcting color/white balance), ML/DL‐based analysis, and final concentration prediction or classification.

Long et al. developed an ML‐assisted, smartphone‐based fluorescence sensing platform for the ultrasensitive detection of chlortetracycline (CTC). A novel fluorescent probe based on AgInS_2_ quantum dots (QDs) enabled visual monitoring of trace levels of CTC, with the fluorescence of the QDs undergoing a blue shift and enhancement due to electrostatic interactions and bandgap transitions, resulting in a visible color change from orange to blue–violet. This platform achieved detection across a wide concentration range, with a limit of detection (LOD) as low as 1.97 nM. To enable quantitative analysis, fluorescence images at different CTC concentrations were converted into RGB color values, generating a data matrix of 300 (RGB features) × 240 (samples) across eight concentrations. An RF algorithm was then applied, with the dataset randomly divided into 168 training samples and 72 testing samples, allowing the RF model to predict CTC concentration accurately [113].

Additionally, ML is increasingly utilized to handle the large and complex datasets generated by array sensors, enabling the reliable identification and quantification of multiple analytes within a single platform. These approaches have been successfully applied to detect a wide range of targets, including antibiotic residues, foodborne pathogens, environmental contaminants, and biomarkers relevant to healthcare. By combining low‐cost sensor platforms with AI‐driven data processing, such systems can achieve enhanced sensitivity, selectivity, and robustness, while maintaining portability and affordability.

Zhu et al. developed an ML‐assisted paper‐based fluorescent sensor array utilizing metal‐doped multicolor CQDs (Ag‐CQDs, Cu‐CQDs, and Zn‐CQDs) as sensing units. The CQDs were patterned directly onto paper substrates using an inkjet printer, allowing for simple fabrication and seamless integration of the sensor array. Owing to the varying affinities of different bacterial species toward the platform, distinct levels of fluorescence quenching were observed. The resulting fluorescence signals were captured using a smartphone, enabling rapid bacterial identification when processed with ML algorithms such as KNN, linear discriminant analysis (LDA), SVM, Naive Bayes classifiers, and decision trees [114]. Xiao et al. developed an ML‐assisted fluorescent sensor array using antibiotic‐modified CQDs, specifically polymyxin B‐CQDs (PM‐CQDs), gentamicin‐CQDs (Gen‐CQDs), and ampicillin‐CQDs (Amp‐CQDs). These functionalized CQDs demonstrated improved sensitivity for detecting foodborne pathogens. The classification was performed using multiple algorithms, including KNN, Naive Bayes, decision trees, LDA, and SVM. To build a reliable dataset, each bacterium was tested in eight replicates with each CQD (3 CQDs × 5 bacteria × 8 replicates). During model development, 70% of the data was allocated for training and 30% for testing, ensuring both robustness and accuracy in bacterial identification [115].

As emphasized in recent reviews on ML‐assisted image‐based optical devices, the main challenge for real‐world translation is often a lack of data rather than the choice of algorithm. Image‐derived features can vary with lighting conditions, distance/angle, exposure, and white balance, device‐to‐device camera differences, compression, and phone‐specific image processing pipelines. Paper‐based devices add further variability through batch‐to‐batch differences in paper substrates and reagent spreading, while humidity and temperature can influence color development. Therefore, reliable deployment benefits from reporting acquisition settings and preprocessing steps [111, 112]. Reviews of image‐based optical devices highlight that translation is frequently limited by domain shift driven by acquisition geometry, illumination, device processing pipelines, and preprocessing choices [116]. Practical smartphone colorimetry studies further demonstrate sensitivity to device and acquisition conditions [112] and to complex ambient lighting [117]. For paper‐based analytical devices, ML‐assisted colorimetric analysis similarly depends on standardized acquisition/preprocessing [118]. Accordingly, reproducible studies should report device and acquisition data (phone model, exposure/white balance, distance/angle, illumination), batch identifiers (paper/reagent), and preprocessing/software versions.

#### 1.2.6. Applications in Drug Stability Studies

AI is increasingly playing a transformative role in drug stability studies, which are critical for ensuring the safety, efficacy, and quality of pharmaceutical products throughout their shelf life. By applying predictive modeling and advanced data analytics, AI enables the accurate prediction of stability profiles, reduces the need for extensive long‐term experiments, lowers costs, and accelerates drug development timelines [119]. An illustrative example is provided by Ajdric et al., who applied a DL approach to predict the stability profile of esomeprazole in a freeze‐dried product. An MLP network with four layers was developed, using pH values of the reconstituted solution and storage time (in months) as inputs. At the same time, the outputs included esomeprazole assay values and four key impurities (4‐hydroxy sulfide impurity, sulfone impurity, 5‐methoxy‐1H‐benzimidazole‐2‐sulfinic acid, and 4‐hydroxy impurity). Data from nine batches, stored at two different temperatures (20°C and −30°C) over 36 months, were used. The data of 81 experimental runs were divided into training, validation, and test sets. The MLP network was trained with a back‐propagation algorithm, and the optimal model was selected based on the lowest RMS error across datasets. This validated model was further applied to establish the design space for the lyophilization process, demonstrating the potential of AI to enhance decision‐making and streamline stability study workflows [120].

## 2. Challenges of AI in Analytical Chemistry

Despite significant progress in applying AI to analytical chemistry, several challenges continue to limit its broader adoption. These challenges can be grouped into data, models, ethics, and regulation.

### 2.1. Data Quality and Availability

- Heterogeneity and complexity: Experimental data from spectroscopy, chromatography, electrochemical sensors, and nanomaterial synthesis are often noisy, high‐dimensional, and inconsistent, complicating model training and validation.
- Scarce datasets: In nanomaterial synthesis and sensor development, limited and inconsistent datasets reduce model generalizability.
- Lack of standardization: Variations in reporting synthesis parameters, instrumental conditions, or preprocessing steps hinder reproducibility and cross‐study comparisons.
- Transferability challenges: Models trained on one instrument, laboratory, or sample matrix often fail to generalize to new conditions, limiting practical deployment.

### 2.2. Model Performance and Reliability

- Overfitting: AI/ML models can fit training data perfectly but fail to predict unseen experimental conditions.
- Underfitting: Oversimplified models may fail to capture the complexity of chemical datasets, resulting in poor predictive performance.
- Limited interpretability: Many ML models act as “black boxes,” restricting the extraction of mechanistic insights. For example, in voltammetric analysis, applying explainable AI methods revealed that peak shifts were primarily due to matrix interferences rather than instrumental drift, which guided better preprocessing and model selection.
- Complexity in model selection: Successful implementation requires careful choice of model type appropriate to dataset size and complexity.

### 2.3. Ethical Challenges

Unethical research practices include the following:

- Use of unlicensed or pirated software tools.
- Data fabrication, such as generating synthetic spectra, chromatograms, or sensor outputs and presenting them as experimental results.
- Lack of transparency in reporting, including selective reporting of favorable results or omitting failed models.
- Autonomous decision‐making risks: AI systems making decisions without human supervision may compromise the reliability and integrity of results.

### 2.4. Regulatory and Reproducibility Challenges

The absence of clear ethical and regulatory frameworks for AI integration in analytical chemistry remains a critical barrier. Black‐box models and inconsistent reporting limit reproducibility and hinder regulatory acceptance, particularly in pharmaceutical or clinical applications. Standardized metrics for model validation, reproducible data, and open‐access datasets are largely lacking.

## 3. Conclusions and Future Perspectives

AI‐based approaches are playing an increasingly important role in modern analytical chemistry. It supports method development and optimization in spectrophotometric and chromatographic workflows, improves interpretation of complex multivariate datasets, and strengthens qualitative and quantitative decisions in challenging matrices. AI can reduce manual trial‐and‐error and enhance throughput, provided that models are trained on representative data and evaluated rigorously. The integration of AI/ML into analytical chemistry offers numerous transformative implications that can significantly enhance research and routine analysis. These technologies enable improved data analysis by facilitating smarter, faster interpretation of complex datasets, which is critical in handling large datasets of analytical results. They also contribute to enhanced detection limits, allowing chemists to identify and quantify trace levels of analytes with greater sensitivity and confidence. Furthermore, AI/ML supports automation techniques that streamline workflows, reduce manual intervention, and minimize human error, leading to increased efficiency and reproducibility.

At the same time, the field is still transitioning from proof‐of‐concept studies to routine laboratory adoption. Major barriers include heterogeneous and nonstandardized datasets, limited transferability, interpretability, and uncertainty for analytical decisions. Ethical and governance considerations, especially privacy, auditability, and transparent reporting, remain critical.

Looking forward, several key research priorities are evident for the next 5–10 years:

• Standardization of data reporting and creation of open‐access, high‐quality datasets to improve reproducibility and enable robust model transfer.
• Development of explainable and interpretable AI models that not only predict outcomes but also provide a mechanistic understanding of chemical processes.
• Integration of AI early in experimental design and method development, particularly for drug stability studies, optimization, and high‐throughput screening.
• Expansion of AI applications in sustainable and green analytical chemistry, including systematic guidance for minimizing hazardous solvent use, energy consumption, and environmental impact while maintaining analytical robustness.
• Bridging regulatory and ethical gaps by establishing transparent, auditable AI workflows suitable for clinical, pharmaceutical, and high‐consequence analytical contexts.
• Addressing the challenges of model generalization, particularly in sensor‐based platforms (e.g., smartphone or paper‐based devices) and nanomaterials, where batch variability and acquisition conditions significantly impact performance.

By focusing on these priorities, AI/ML can evolve from proof‐of‐concept studies to reliable, reproducible, and sustainable tools, advancing both analytical performance and ethical research practices. Collectively, these strategies will enhance the reliability of results, reinforce the ethical foundation of analytical research, and promote a more sustainable and forward‐looking future for AI‐based analytical chemistry.

## Funding

The authors have nothing to report.

## Ethics Statement

The authors have nothing to report.

## Conflicts of Interest

The authors declare no conflicts of interest.

## Data Availability

All data in this review are included in this published article.

## References

[1] Chowdhury I. J. , Hossan M. I. , Khan M. T. R. et al., Revolutionizing Drug Discovery: A Systematic Review of AI and Machine Learning Application, *2025 3rd International Conference on Self Sustainable Artificial Intelligence Systems (ICSSAS)*, 2025, 1814–1821, 10.1109/ICSSAS66150.2025.11081368.

[2] Deng J. , Yang Z. , Ojima I. , Samaras D. , and Wang F. , Artificial Intelligence in Drug Discovery: Applications and Techniques, Briefings in Bioinformatics. (2022) 23, no. 1, 10.1093/bib/bbab430. 34734228

[3] Blanco-Gonzalez A. , Cabezon A. , Seco-Gonzalez A. et al., The Role of AI in Drug Discovery: Challenges, Opportunities, and Strategies, Pharmaceuticals. (2023) 16, no. 6, 10.3390/ph16060891. PMC10302890 37375838

[4] Chopra H. , Baig A. A. , Gautam R. K. , and Kamal M. A. , Application of Artificial Intelligence in Drug Discovery, Current Pharmaceutical Design. (2022) 28, no. 33, 2690–2703, 10.2174/1381612828666220608141049. 35676841

[5] Walton N. A. , Nagarajan R. , Wang C. et al., Others, Enabling the Clinical Application of Artificial Intelligence in Genomics: a Perspective of the AMIA Genomics and Translational Bioinformatics Workgroup, Journal of the American Medical Informatics Association. (2024) 31, no. 2, 536–541, 10.1093/jamia/ocad211. 38037121 PMC10797281

[6] Dara M. , Dianatpour M. , Azarpira N. , and Tanideh N. , The Transformative Role of Artificial Intelligence in Genomics: Opportunities and Challenges, Gene Reports. (2025) 41, 10.1016/j.genrep.2025.102314.

[7] Al Souleiman I. and Al Souleiman H. , AI-driven Genomic Medicine: A Comprehensive Review of Clinical Applications, Institutional Dynamics, and Governance Challenges, Intelligence-Based Medicine. (2026) 14, 10.1016/j.ibmed.2026.100365.

[8] Li S. , Fan W. , and Zhou Y. , AI-empowered Genome Decoding: Applications of Large Language Models in Genomics, Front. Digit. Educ.(2025) 2, no. 1, 10.3389/feduc.2025.1533072.

[9] Srivastava R. , Advancing Precision Oncology with AI-powered Genomic Analysis, Frontiers in Pharmacology. (2025) 16, 10.3389/fphar.2025.1591696. PMC12075946 40371349

[10] Nagarajan R. , Wang C. , Walton D. , and Walton N. , Artificial Intelligence Applications in Genomics, Advances in Molecular Pathology. (2024) 7, no. 1, 145–154, 10.1016/j.yamp.2024.07.003.

[11] Gangwal A. and Lavecchia A. , Artificial Intelligence in Natural Product Drug Discovery: Current Applications and Future Perspectives, Journal of Medicinal Chemistry. (2025) 68, no. 4, 3948–3969, 10.1021/acs.jmedchem.4c02873. 39916476 PMC11874025

[12] Chen W. , Liu X. , Zhang S. , and Chen S. , Artificial Intelligence for Drug Discovery: Resources, Methods, and Applications, Molecular Therapy Nucleic Acids. (2023) 31, 691–702, 10.1016/j.omtn.2023.02.019. 36923950 PMC10009646

[13] Murphy S. , The Role of Artificial Intelligence in Enhancing Analytical Chemistry Techniques to Streamline Drug Development and Testing Processes, Journal of Analytical & Bioanalytical Techniques. (2025) 16, 10.4172/2155-9872.1000752.

[14] Jayalelshmi S. , Farrell J. , Chen Y. , Vargas Izquierdo M. F. , and Makarychev-Mikhailov S. M. , Can Artificial Intelligence Design Your Experiment? A Carbon Capture Example, *SPE International Conference on Oilfield Chemistry*, 2025, 10.2118/223475-MS.

[15] Manepally A. , Narimela M. , Puli S. , Meesa M. , and Daram S. R. , Others, Applications and Tools of Artificial Intelligence in Analytical Method Development by HPLC, International Journal of Science and Research Archive. (2025) 15, 811–826, 10.30574/ijsra.2025.15.1.0108.

[16] Abba S. I. , Usman A. G. , and Işik S. , Others, Simulation for Response Surface in the HPLC Optimization Method Development Using Artificial Intelligence Models: A Data-Driven Approach, Chemometrics and Intelligent Laborary Systems. (2020) 201, 10.1016/j.chemolab.2020.104007.

[17] Rahman S. N. R. , Katari O. , Pawde D. M. et al., Application of Design of experiments®approach-driven Artificial Intelligence and Machine Learning for Systematic Optimization of Reverse Phase High Performance Liquid Chromatography Method to Analyze Simultaneously Two Drugs (Cyclosporin A and Etodolac), AAPS PharmSciTech. (2021) 22, no. 4, 10.1208/s12249-021-02017-3. 33987739

[18] Singh Y. R. , Shah D. B. , Kulkarni M. et al., Current Trends in Chromatographic Prediction Using Artificial Intelligence and Machine Learning, Analytical Methods. (2023) 15, no. 23, 2785–2797, 10.1039/D3AY00362K. 37264667

[19] Barseem A. , Obaydo R. H. , and Elagamy S. H. , Smartphone-Based Colorimetric Determination of Imeglimin Hydrochloride Using Eosin Y: a Simple and Eco-Friendly Analytical Approach, Analytical Science Advances. (2025) 6, no. 2, 10.1002/ansa.70064. PMC12746043 41473335

[20] Elagamy S. H. , Fuente-Ballesteros A. , Hasan Obaydo R. , and Mahmoud Lotfy H. , Eco-Friendly and Portable Sensing: a Review of Advances in Smartphone-Integrated Optical Nanoprobes, Green Chemistry Letters and Reviews. (2025) 18, no. 1, 10.1080/17518253.2025.2548507.

[21] Hammad S. F. , Hamid M. A. A. , Elagamy S. H. , and Adly L. , Smartphone-Assisted HPTLC for Simultaneous Determination of Vonoprazan Fumarate and Aspirin: a Comparative Study with HPTLC Densitometry, Scientific Reports. (2025) 10.1038/s41598-025-93043-z. PMC12647887 41286293

[22] Elagamy S. H. , Amin K. F. M. , Obaydo R. H. , and Lotfy H. M. , Review of Green Synthesized Carbon Dots: Applications in Pharmaceutical Drug Sensing, Talanta Open. (2025) 12, 10.1016/j.talo.2025.100579.

[23] Elagamy S. H. , Obaydo R. H. , Barseem A. , and Lotfy H. , Dual Emissive Carbon Dots as Ratiometric Fluorescence Sensors: a Comprehensive Review, Sens. Bio-Sensing Res.(2025) 50, 10.1016/j.sbsr.2025.100930.

[24] Barseem A. , Obaydo R. H. , and Elagamy S. H. , Bio-Inspired Linseed Derived Carbon Quantum Dots as a Fluorescent Probe for the Fluorimetric Determination of Imeglimin in Biological Fluids and Dosage Forms, Sens. Bio-Sensing Res.(2025) 51, 10.1016/j.sbsr.2025.100946.

[25] Barseem A. , Elshahawy M. , and Elagamy S. H. , Fluorimetric Determination of Vonoprazan via Quenching of Nitrogen and Sulfur co-doped Carbon Quantum Dots: a Rapid and Sustainable Analytical Approach, Luminescence. (2024) 39, no. 7, 10.1002/bio.4834. 39036968

[26] Ozer T. and Henry C. S. , Recent Advances in Sensor Arrays for the Simultaneous Electrochemical Detection of Multiple Analytes, Journal of the Electrochemical Society. (2021) 168, 10.1149/1945-7111/abf523.

[27] Bao G.-M. , Chen D.-D. , Xia Y.-F. et al., Single-Well Colorimetric Sensor Array for Discrimination and smartphone-Assisted Detection of Catecholamines Based on Fe-Carbon Dots Nanozymes, Analytica Chimica Acta. (2025) 1355, 10.1016/j.aca.2025.343997. 40274328

[28] Yang X. , Li Q. , Kwee S. , Yang J. , Zhang Q. , and Hu X. , An Immunochromatographic Strip Sensor for Marbofloxacin Residues, PLoS One. (2024) 19, no. 3, 10.1371/journal.pone.0299709. PMC10980191 38551994

[29] Cancelliere R. , Molinara M. , Licheri A. , Maffucci A. , and Micheli L. , Artificial intelligence-assisted Electrochemical Sensors for Qualitative and Semi-quantitative Multiplexed Analyses, Digestive Diseases. (2025) 4, no. 2, 338–342, 10.1039/D4DD00321G.

[30] Rial R. C. , AI in Analytical Chemistry: Advancements, Challenges, and Future Directions, Talanta. (2024) 274, 10.1016/j.talanta.2024.125949. 38569367

[31] Debus B. , Parastar H. , Harrington P. , and Kirsanov D. , Deep Learning in Analytical Chemistry, TrAC, Trends in Analytical Chemistry. (2021) 145, 10.1016/j.trac.2021.116459.

[32] Houhou R. and Bocklitz T. , Trends in Artificial Intelligence, Machine Learning, and Chemometrics Applied to Chemical Data, Anal. Sci. Adv. (2021) 2, no. 3-4, 128–141, 10.1002/ansa.202000162. 38716450 PMC10989568

[33] Naidu G. , Zuva T. , and Sibanda E. M. , A Review of Evaluation Metrics in Machine Learning Algorithms, Lecture Notes in Networks and Systems, *Computer Science On-line Conference*, 2023, 15–25, 10.1007/978-3-031-35314-7_2.

[34] Sarker I. H. , Machine Learning: Algorithms, real-world Applications and Research Directions, SN Computer Science. (2021) 2, no. 3, 10.1007/s42979-021-00592-x. PMC7983091 33778771

[35] Binson V. A. , Thomas S. , Subramoniam M. , Arun J. , Naveen S. , and Madhu S. , A Review of Machine Learning Algorithms for Biomedical Applications, Annals of Biomedical Engineering. (2024) 52, no. 5, 1159–1183, 10.1007/s10439-024-03459-3. 38383870

[36] Naeem S. , Ali A. , Anam S. , and Ahmed M. M. , An Unsupervised Machine Learning Algorithms: Comprehensive Review, Int. J. Comput. Digit. Syst.(2023) 13, no. 1, 911–921, 10.12785/ijcds/130172.

[37] Obaido G. , Mienye I. D. , Egbelowo O. F. et al., Supervised Machine Learning in Drug Discovery and Development: Algorithms, Applications, Challenges, and Prospects, Machine Learning with Application. (2024) 17, 10.1016/j.mlwa.2024.100576.

[38] Janiesch C. , Zschech P. , and Heinrich K. , Machine Learning and Deep Learning, Electronic Markets. (2021) 31, no. 3, 685–695, 10.1007/s12525-021-00475-2.

[39] Abdel-Jaber H. , Devassy D. , Al Salam A. , Hidaytallah L. , and El-Amir M. , A Review of Deep Learning Algorithms and Their Applications in Healthcare, Algorithms. (2022) 15, no. 2, 10.3390/a15020071.

[40] Waqas M. , Naseem A. , Humphries U. W. , Hlaing P. T. , Dechpichai P. , and Wangwongchai A. , Applications of Machine Learning and Deep Learning in Agriculture: A Comprehensive Review, Green Technol, Sustainable Times. (2025) 3, 10.1016/j.grets.2025.100199.

[41] Guo S. , Popp J. , and Bocklitz T. , Chemometric Analysis in Raman Spectroscopy from Experimental Design to Machine Learning–based Modeling, Nature Protocols. (2021) 16, no. 12, 5426–5459, 10.1038/s41596-021-00624-3. 34741152

[42] Puthongkham P. , Wirojsaengthong S. , and Suea-Ngam A. , Machine Learning and Chemometrics for Electrochemical Sensors: Moving Forward to the Future of Analytical Chemistry, Analyst. (2021) 146, no. 21, 6351–6364, 10.1039/D1AN01148K. 34585185

[43] Joshi P. B. , Navigating with Chemometrics and Machine Learning in Chemistry, Artificial Intelligence Review. (2023) 56, no. 9, 9089–9114, 10.1007/s10462-023-10391-0. PMC9870782 36714038

[44] Nandan M. , Mitra S. , and De D. , GraphXAI: a Survey of Graph Neural Networks (GNNs) for Explainable AI (XAI), Neural Computing & Applications. (2025) 37, no. 17, 1–52, 10.1007/s00521-025-11098-7.

[45] Bugueño M. , Biswas R. , and de Melo G. , Graph-Based Explainable AI: A Comprehensive Survey, 2024, 10.48550/arXiv.2404.17065.

[46] Makumbura R. K. , Mampitiya L. , Rathnayake N. et al., Advancing Water Quality Assessment and Prediction Using Machine Learning Models, Coupled with Explainable Artificial Intelligence (XAI) Techniques like Shapley Additive Explanations (SHAP) for Interpreting the black-box Nature, Results in Engineering. (2024) 23, 10.1016/j.rineng.2024.102831.

[47] Zhong X. , Gallagher B. , Liu S. , Kailkhura B. , Hiszpanski A. , and Han T. Y.-J. , Explainable Machine Learning in Materials Science, npj Computational Materials. (2022) 8, no. 1, 10.1038/s41524-022-00884-7.

[48] Oviedo F. , Ferres J. L. , Buonassisi T. , and Butler K. T. , Interpretable and Explainable Machine Learning for Materials Science and Chemistry, Accounts of Materials Research. (2022) 3, no. 6, 597–607, 10.1021/accountsmr.1c00244.

[49] Belle V. and Papantonis I. , Principles and Practice of Explainable Machine Learning, Frontiers in Big Data. (2021) 4, 10.3389/fdata.2021.688969. PMC8281957 34278297

[50] Meza Ramirez C. A. , Greenop M. , Ashton L. , and Rehman I. U. , Applications of Machine Learning in Spectroscopy, Applied Spectroscopy Reviews. (2021) 56, no. 8-10, 733–763, 10.1080/05704928.2020.1859525.

[51] Fu W. and Hopkins W. S. , Applying Machine Learning to Vibrational Spectroscopy, Journal of Physical Chemistry A. (2018) 122, no. 1, 167–171, 10.1021/acs.jpca.7b10303. 29211476

[52] Madden M. G. and Howley T. , A Machine Learning Application for Classification of Chemical Spectra, *Applications and Innovations in Intelligent Systems XVI*, 2008, 77–90, 10.1007/978-1-84882-215-3_6.

[53] Aoyagi S. , Application of Machine Learning to Spectrum and Image Data, Journal of Vacuum Science and Technology A. (2023) 41, 10.1116/6.0002661.

[54] Goodacre R. , Explanatory Analysis of Spectroscopic Data Using Machine Learning of Simple, Interpretable Rules, Vibrational Spectroscopy. (2003) 32, no. 1, 33–45, 10.1016/S0924-2031(03)00045-6.

[55] Razavi R. and Kenari R. E. , Ultraviolet–Visible Spectroscopy Combined with Machine Learning as a Rapid Detection Method to the Predict Adulteration of Honey, Heliyon. (2023) 9, no. 10, 10.1016/j.heliyon.2023.e14457. PMC10597822 37886742

[56] Gu H.-W. , Zhou H.-H. , Lv Y. et al., Geographical Origin Identification of Chinese Red Wines Using ultraviolet-visible Spectroscopy Coupled with Machine Learning Techniques, Journal of Food Composition and Analysis. (2023) 119, 10.1016/j.jfca.2023.105265.

[57] Mamede R. , Pereira F. , and Aires-de-Sousa J. , Machine Learning Prediction of UV–Vis Spectra Features of Organic Compounds Related to Photoreactive Potential, Scientific Reports. (2021) 11, no. 1, 10.1038/s41598-021-03070-9. PMC8660842 34887473

[58] Franca T. , Goncalves D. , and Cena C. , ATR-FTIR Spectroscopy Combined with Machine Learning for Classification of PVA/PVP Blends in Low Concentration, Vibrational Spectroscopy. (2022) 120, 10.1016/j.vibspec.2022.103378.

[59] Enyoh C. E. and Wang Q. , Automated Classification of Undegraded and Aged Polyethylene Terephthalate Microplastics from ATR-FTIR Spectroscopy Using Machine Learning Algorithms, Journal of Polymers and the Environment. (2024) 32, no. 9, 4143–4158, 10.1007/s10924-024-03200-w.

[60] Chang K. H. and Chua H. N. , A Machine Learning Model for the Classification of Illicit Drug Substances with Fourier Transform Infrared Spectroscopy, Microchemical Journal. (2025) 212, 10.1016/j.microc.2025.113427.

[61] Calle J. L. P. , Ferreiro-González M. , Ruiz-Rodríguez A. , Fernández D. , and Palma M. , Detection of Adulterations in Fruit Juices Using Machine Learning Methods over FT-IR Spectroscopic Data, Agronomy. (2022) 12, no. 3, 10.3390/agronomy12030683.

[62] Lotfy H. M. , Obaydo R. H. , and Mouhamed A. A. , GLANCE Visualization for Smart Analytical Chemistry Methods: Artificial Intelligence for Spectrophotometric Determination of Solifenacin-Mirabegron Combination, Sustainable Chemistry and Pharmacy. (2025) 47, 10.1016/j.scp.2025.102159. PMC13134955 42067525

[63] Klukowski P. , Riek R. , and Güntert P. , Machine Learning in NMR Spectroscopy, Progress in Nuclear Magnetic Resonance Spectroscopy. (2025) 148-149, 10.1016/j.pnmrs.2025.101575. 40912880

[64] Zhang W. , Kasun L. C. , Wang Q. J. , Zheng Y. , and Lin Z. , A Review of Machine Learning for Near-Infrared Spectroscopy, Sensors. (2022) 22, no. 24, 10.3390/s22249764. PMC9784128 36560133

[65] Song Y. , Yi W. , Liu Y. , Zhang C. , Wang Y. , and Ning J. , A Robust Deep Learning Model for Predicting Green Tea Moisture Content During Fixation Using near-infrared Spectroscopy: Integration of multi-scale Feature Fusion and Attention Mechanisms, Food Research International. (2025) 203, 10.1016/j.foodres.2025.115874. 40022390

[66] Wang X. , Wu H. , Wang T. et al., NIRFluor: a Deep Learning Platform for Rapid Screening of Small Molecule Near-Infrared Fluorophores with Desired Optical Properties, Analytical Chemistry. (2025) 97, no. 4, 1992–2002, 10.1021/acs.analchem.4c05412. 39818744

[67] Xiao T. , Xie C. , Yang L. et al., A General Deep Learning Model for Predicting and Classifying Pea Protein Content via Visible and near-infrared Spectroscopy, Food Chemistry. (2025) 478, 10.1016/j.foodchem.2025.143617. 40049135

[68] Qi Y. , Hu D. , Jiang Y. et al., Recent Progresses in Machine Learning Assisted Raman Spectroscopy, Advanced Optical Materials. (2023) 11, no. 14, 10.1002/adom.202203104.

[69] Domingos C. , Fantoni A. , Fernandes M. , Fidalgo J. , and Pereira S. A. , Low-Cost Raman Spectroscopy Setup Combined with a Machine Learning Model, Sensors. (2025) 25, no. 3, 10.3390/s25030659. PMC11821046 39943297

[70] Lee J. U. and Kim H. J. , Integration of Machine Learning in Surface-Enhanced Raman Spectroscopy Biosensor for Biomedical Applications, Biochip Journal. (2025) 19, no. 3, 1–12, 10.1007/s13206-024-00186-0.

[71] Chen J. , Xiong X. , Ye J. et al., Machine learning-assisted three-dimensional Fluorescence for Heavy Metal Multi-Sensing, Sensors and Actuators B: Chemical. (2025) 431, 10.1016/j.snb.2025.137385.

[72] Zhang Q. , Li X. , Yu L. et al., Machine learning-assisted Fluorescence Visualization for Sequential Quantitative Detection of Aluminum and Fluoride Ions, Journal of Environmental Sciences. (2025) 149, 68–78, 10.1016/j.jes.2024.01.042. 39181678

[73] Hategan A. R. , Pirnau A. , and Magdas D. A. , Applications of Machine Learning for Wine Recognition Based on 1H-NMR Spectroscopy, Beverages. (2025) 11, no. 2, 10.3390/beverages11020045.

[74] Günaydin S. , Çetin N. , Saglam C. , Sacilik K. , and Jahanbakhshi A. , Comparative Analysis of Visible and near-infrared (Vis-NIR) Spectroscopy and Prediction of Moisture Ratio Using Machine Learning Algorithms for Jujube Dried Under Different Conditions, Applied Food Research. (2025) 5, no. 1, 10.1016/j.afres.2025.100699.

[75] Zhou Y. , Tang X. , Zhang D. , and Lee H. J. , Machine Learning Empowered Coherent Raman Imaging and Analysis for Biomedical Applications, Communications Engineer. (2025) 4, no. 1, 10.1038/s44172-025-00356-8. PMC11761466 39856240

[76] Tang J.-W. , Yuan Q. , Zhang L. , Marshall B. J. , Tay A. C. Y. , and Wang L. , Application of Machine learning-assisted surface-enhanced Raman Spectroscopy in Medical Laboratories: Principles, Opportunities, and Challenges, TrAC, Trends in Analytical Chemistry. (2025) 184, 10.1016/j.trac.2025.118135.

[77] Sarmanova O. E. , Kudryashov A. D. , Laptinskiy K. A. et al., Applications of Fluorescence Spectroscopy and Machine Learning Methods for Monitoring of Elimination of Carbon Nanoagents from the Body, Optical Memory & Neural Networks. (2023) 32, no. 1, 20–30, 10.3103/S1060992X23050089.

[78] Post C. , Brülisauer S. , Waldschläger K. et al., Application of Laser-Induced, Deep Uv Raman Spectroscopy and Artificial Intelligence in real-time Environmental Monitoring—Solutions and First Results, Sensors. (2021) 21, no. 11, 10.3390/s21113911. PMC8201312 34198916

[79] Srivastava S. , Wang W. , Zhou W. , Jin M. , and Vikesland P. J. , Machine Learning-Assisted Surface-Enhanced Raman Spectroscopy Detection for Environmental Applications: A Review, Environmental Science and Technology. (2024) 58, 20830–20848, 10.1021/acs.est.4c06195. 39537382 PMC11603787

[80] Zhou F. , Chu J. , Lu F. , Ouyang W. , Liu Q. , and Wu Z. , Real-Time Monitoring of Methyl Orange Degradation in Non-thermal Plasma by Integrating Raman Spectroscopy with a Hybrid Machine Learning Model, Environmental Technology & Innovation. (2025) 38, 10.1016/j.eti.2025.104100.

[81] Lee Y. J. , Jeong C. W. , Kim H. T. , Lee T.-J. , and Kim H. J. , Raman Spectroscopy and Machine Learning for Forensic Document Examination, Analyst. (2025) 150, no. 9, 1785–1794, 10.1039/D4AN01613K. 40168015

[82] Gouzou D. , Taimori A. , Haloubi T. et al., Applications of Machine Learning in time-domain Fluorescence Lifetime Imaging: a Review, Methods and Applications in Fluorescence. (2024) 12, no. 2, 10.1088/2050-6120/ad2f98. PMC10851337 38055998

[83] Shchechkin I. D. , Recent Trends of Fluorescence Lifetime Imaging Microscopy Analysis Using Machine Learning, J. Biomed. Photonics & Eng.(2025) 11, 4–22, 10.18287/JBPE25.11.010101.

[84] Wang F. , Li J. , Wang F. et al., Machine Learning-based Predictive Modeling of HAAs Concentration in Secondary Water Supply System Using UV–vis Absorption and excitation-emission Matrix (EEM) Fluorescence Spectroscopy, Microchemical Journal. (2025) 215, 10.1016/j.microc.2025.114329.

[85] Kalatzis D. , Nega A. , and Kiouvrekis Y. , Raman Spectra Classification of Pharmaceutical Compounds: a Benchmark of Machine Learning Models with SHAP-Based Explainability, Engage. (2025) 6, no. 7, 10.3390/eng6070145.

[86] Rahman M. S. , Khan M. K. H. , Kawakami J. , Wang K. , Diaz A. , and Riley F. , Recent Applications of Liquid Chromatography-based QSRR Models for Pharmaceutically Relevant Small Molecules: a Review, Journal of Pharmaceutical Sciences. (2026) 115, no. 1, 10.1016/j.xphs.2025.104047. 41176062

[87] Usman A. G. , Işik S. , and Abba S. I. , Qualitative Prediction of Thymoquinone in the high-performance Liquid Chromatography Optimization Method Development Using Artificial Intelligence Models Coupled with Ensemble Machine Learning, Separation Science Plus. (2022) 5, no. 10, 579–587, 10.1002/sscp.202200069.

[88] Kensert A. , Desmet G. , and Cabooter D. , Separation Science: the State of the Art: Graph Neural Networks for Improved Retention Time Predictions, Wiley-VCH. (2022) 10.1002/9783527836444.ch13.

[89] Malarvannan M. , Kumar K. V. , Reddy Y. P. et al., Assessment of Computational Approaches in the Prediction of Spectrogram and Chromatogram Behaviours of Analytes in Pharmaceutical Analysis: Assessment Review, Future Journal of Pharmaceutical Sciences. (2023) 9, no. 1, 10.1186/s43094-023-00536-9.

[90] Lotfy H. M. , Erk N. , Genc A. A. , Obaydo R. H. , and Tiris G. , Artificial Intelligence in Chromatography: Greenness and Performance Evaluation of AI-Predicted Versus In-Lab Optimized HPLC Methods for Simultaneous Separation of Amlodipine, Hydrochlorothiazide, and Candesartan, Talanta Open. (2025) 11, 10.1016/j.talo.2025.100473.

[91] Do T. K. T. , Trettin I. , Hänni M. , and Reich E. , Applying Machine Learning on high-performance thin-layer Chromatography Using the Complementary Developing Solvents Concept, Journal of Liquid Chromatography & Related Technologies. (2025) 48, 111–117, 10.1080/10826076.2025.2460949.

[92] Laganà Vinci R. , Arena K. , Rigano F. et al., Quantitative Structure Retention Relationship Applied to Capillary Scale Liquid Chromatography for the Identification of Phenolic Compounds, Green Anal. Chem.(2026) 16, 10.1016/j.greeac.2025.100321.

[93] Singh Y. R. , Shah D. B. , Maheshwari D. G. , Shah J. S. , and Shah S. , Advances in AI-Driven Retention Prediction for Different Chromatographic Techniques: Unraveling the Complexity, Critical Reviews in Analytical Chemistry. (2024) 54, no. 8, 3559–3569, 10.1080/10408347.2023.2254379. 37672314

[94] Kumar M. , Nandi A. , Yadav R. L. , Das Gupta G. , and Sharma K. , AI-Enhanced Prediction Tools and Sensor Integration in Advanced Analytical Chemistry Techniques, Current Analytical Chemistry. (2025) 10.2174/0115734110339498250114114400.

[95] Ciura K. , Fryca I. , and Gromelski M. , Prediction of the Retention Factor in Cetyltrimethylammonium Bromide Modified Micellar Electrokinetic Chromatography Using a Machine Learning Approach, Microchemical Journal. (2023) 187, 10.1016/j.microc.2023.108393.

[96] Fedorova E. S. , Matyushin D. D. , Plyushchenko I. V. , Stavrianidi A. N. , and Buryak A. K. , Deep Learning for Retention Time Prediction in Reversed-phase Liquid Chromatography, Journal of Chromatography, A. (2022) 1664, 10.1016/j.chroma.2021.462792. 34999303

[97] Szucs R. , Brown R. , Brunelli C. , Heaton J. C. , and Hradski J. , Structure Driven Prediction of Chromatographic Retention Times: Applications to Pharmaceutical Analysis, International Journal of Molecular Sciences. (2021) 22, no. 8, 10.3390/ijms22083848. PMC8068189 33917733

[98] Krmar J. , Svrkota B. , Đajić N. , Stojanović J. , Protić A. , and Otašević B. , QSRR Approach: Application to Retention Mechanism in Liquid Chromatography, Nov. Asp. Gas Chromatogr. Chemom.(2023) 113–141, 10.5772/intechopen.106245.

[99] Moorthy N. S. H. N. , Baghel S. S. , Porte N. , Hakkeem S. , Tiwari A. , and Karthikeyan C. , Machine Learning-based Analysis on Retention Time of Pesticides for Ecofriendly and Cost-Effective Analytical Method Development, Silico Res. Biomed. (2025) 1, 10.1016/j.isrb.2025.100050.

[100] Shi Z. , Yi Y. , Madrigal E. , Hrovat F. , Zhang K. , and Lin J. , A Generalizable Methodology for Predicting Retention Time of Small Molecule Pharmaceutical Compounds Across Reversed-phase HPLC Columns, Journal of Chromatography, A. (2025) 1742, 10.1016/j.chroma.2024.465628. 39798480

[101] Molinara M. , Cancelliere R. , Di Tinno A. et al., A Deep Learning Approach to Organic Pollutants Classification Using Voltammetry, Sensors. (2022) 22, no. 20, 10.3390/s22208032. PMC9608622 36298383

[102] Dean S. N. , Shriver-Lake L. C. , Stenger D. A. , Erickson J. S. , Golden J. P. , and Trammell S. A. , Machine Learning Techniques for Chemical Identification Using Cyclic Square Wave Voltammetry, Sensors. (2019) 19, no. 10, 10.3390/s19102392. PMC6567068 31130606

[103] Sharmila V. G. , Kumar M. D. , and Tamilarasan K. , Machine Learning-Driven Advances in Metal-Organic Framework Nanomaterials for Wastewater Treatment: Developments and Challenges, Separation and Purification Reviews. (2024) 1–21, 10.1080/15422119.2024.2434053.

[104] Price C. C. , Li Y. , Zhou G. et al., Predicting and Accelerating Nanomaterial Synthesis Using Machine Learning Featurization, Nano Letters. (2024) 24, no. 46, 14862–14867, 10.1021/acs.nanolett.4c03781. 39529538

[105] Tripathy A. , Patne A. Y. , Mohapatra S. , and Mohapatra S. S. , Convergence of Nanotechnology and Machine Learning: the State of the Art, Challenges, and Perspectives, International Journal of Molecular Sciences. (2024) 25, no. 22, 10.3390/ijms252212368. PMC11594285 39596433

[106] Pashkov D. M. , Guda A. A. , Kirichkov M. V. et al., Quantitative Analysis of the UV–vis Spectra for Gold Nanoparticles Powered by Supervised Machine Learning, Journal of Physical Chemistry C. (2021) 125, no. 16, 8656–8666, 10.1021/acs.jpcc.0c11380.

[107] Salahinejad M. and Roozbahani A. , Applications of Machine Learning Predictive Modeling for Carbon Quantum Dots, Mater. Informatics II Softw. Tools Databases, 2025, Springer, 81–108, 10.1007/978-3-031-81828-3_4.

[108] Mohammadpoor M. , Others, Machine Learning-Driven Approaches for Synthesizing Carbon Dots and Their Applications in Photoelectrochemical Sensors, Inorganic Chemistry Communications. (2024) 159, 10.1016/j.inoche.2023.111859.

[109] Kannouma R. E. , Kamal A. H. , Hammad M. A. , and Mansour F. R. , Machine Learning and Genetic Algorithm Prediction of High Quantum Yield Carbon Quantum Dots for Chemical Analysis, Microchemical Journal. (2025) 208, 10.1016/j.microc.2024.112499.

[110] Ayres L. B. , Gomez F. J. V. , Linton J. R. , Silva M. F. , and Garcia C. D. , Taking the Leap Between Analytical Chemistry and Artificial Intelligence: a Tutorial Review, Analytica Chimica Acta. (2021) 1161, 10.1016/j.aca.2021.338403. 33896558

[111] Sharma P. , Beery D. , Crutchfield A. et al., Huetools: a Modular Image Processing and ML Toolkit for Quantitative Colorimetric Assay Development, SLAS Technol. (2026) 38, 10.1016/j.slast.2026.100413. 41864342

[112] Yüzer E. , Doğan V. , Kılıç V. , and Şen M. , Smartphone Embedded Deep Learning Approach for Highly Accurate and Automated Colorimetric Lactate Analysis in Sweat, Sensors and Actuators B: Chemical. (2022) 371, 10.1016/j.snb.2022.132489.

[113] Long W. , Lu H. , Han Y. et al., Machine learning-assisted Smartphone-based Fluorescence Visual Sensing Platform for Ultrasensitive Detection of Chlortetracycline, Sensors and Actuators B: Chemical. (2025) 428, 10.1016/j.snb.2025.137241.

[114] Zhu L. , Mei L. , Xuan Y. , and Wang F. , Machine Learning Assisted Paper-based Fluorescent Sensor Array with metal-doped Multicolor Carbon Quantum Dots for Identification and Inactivation of Bacteria, Talanta. (2025) 293, 10.1016/j.talanta.2025.128035. 40187285

[115] Xiao M. , Mei L. , Qi J. , Zhu L. , and Wang F. , Functionalized Carbon Quantum Dots Fluorescent Sensor Array Assisted by a Machine Learning Algorithm for Rapid Foodborne Pathogens Identification, Microchemical Journal. (2024) 201, 10.1016/j.microc.2024.110701.

[116] Mousavizadegan M. , Shalileh F. , Mostajabodavati S. , Mohammadi J. , and Hosseini M. , Machine learning-assisted Image-based Optical Devices for Health Monitoring and Food Safety, TrAC, Trends in Analytical Chemistry. (2024) 177, 10.1016/j.trac.2024.117794.

[117] Bao X. , Jiang S. , Wang Y. , Yu M. , and Han J. , A Remote Computing Based point-of-care Colorimetric Detection System with a Smartphone Under Complex Ambient Light Conditions, Analyst. (2018) 143, no. 6, 1387–1395, 10.1039/C7AN01898C. 29451280

[118] Khanal B. , Pokhrel P. , Khanal B. , and Giri B. , Machine-Learning-Assisted Analysis of Colorimetric Assays on Paper Analytical Devices, ACS Omega. (2021) 6, no. 49, 33837–33845, 10.1021/acsomega.1c05191. 34926930 PMC8675014

[119] Jarallah S. J. , Almughem F. A. , Alhumaid N. K. et al., Artificial Intelligence Revolution in Drug Discovery: a Paradigm Shift in Pharmaceutical Innovation, International Journal of Pharmaceutics. (2025) 680, 10.1016/j.ijpharm.2025.125789. 40451590

[120] Ajdarić J. , Ibrić S. , Pavlović A. , Ignjatović L. , and Ivković B. , Prediction of Drug Stability Using Deep Learning Approach: Case Study of Esomeprazole 40 mg Freeze-Dried Powder for Solution, Pharmaceutics. (2021) 13, no. 6, 10.3390/pharmaceutics13060829. PMC8230350 34204912

